# Exploratory Laparatomy—Pregnant Uterus

**Published:** 1893-08

**Authors:** Matthew D. Mann

**Affiliations:** Buffalo, N. Y.


### EXPLORATORY LAPARATOMY—PREGNANT UTERUS.

By MATTHEW D. MANN, M. D., Buffalo, N. Y.

Delivered at the Buffalo General Hospital, February 16, 1891, (Reported by A. L. BENEDICT, M. D.)

A patient was brought to me last week from out of town, the physician stating that while he did not know exactly what the condition was, he was quite sure that laparatomy ought to be done at once. He thought so from the severe pelvic pain that the patient suffered, from numerous hysterical and nervous symptoms which prevailed, and from a long history of invalidism. I

examined the patient without being able to satisfy myself as to the nature of her trouble, and I told the doctor and the patient's friends that the only way to reach a certain diagnosis was by examination under ether. With their consent, I made a careful examination, and even then I was not able to recognize very much, but it seemed to me that right in front of the cervix was a soft cystic mass. I was very much at a loss to account for this. It did not seem to be anything inside the uterus, for the cervix had been dilated forcibly within a short time, the cavity had been curetted, the sound had been passed, and every evidence had showed that the uterus was empty. The cervix was hard and firm, the patient was flowing somewhat at the time, and she had menstruated only a short time before. I told the friends simply that I could not determine what the condition was.

As the symptoms were very severe and the friends and the patient herself were very urgent, I told her that I would make an exploratory operation. Last Tuesday I opened the abdomen, and when I did so, I came down upon a mass, a cyst apparently, which had neither the color nor the appearance of an ovarian cyst, and on pulling the omentum up over it, it struck me at once that it was the uterus. I therefore enlarged the incision sufficiently to admit my whole hand, and, to my astonishment, I found that the woman was pregnant, and that I held in my hand a pregnant uterus of the fourth month. I recognized this by passing my hand around the uterus; but even when I had the abdomen open and the uterus in my hand, it was so soft and placid, and apparently so thin-walled, that it was difficult to recognize it. It did not feel much more consistent than intestine. It was owing to the extreme placidity that I had not recognized it by bimanual palpation even with the patient under ether.

Having recognized the condition, there was nothing to do but close the wound, and the woman had not much more reaction than if I had not done anything. She had no elevation of temperature until Sunday night, when it suddenly jumped up to 106 degrees without apparent cause. She had a violent chill and then she began to have pains, and about nine o'clock the house-physician telephoned that the fetus had come away. I came over and found the os fully dilated, with the placenta adherent. I therefore got it out with placenta forceps and washed out the uterus with a sublimate solution. The management of the abortion was a little complicated by the tenderness of the abdomen, due to the laparatomy. It was utterly impossible to make bimanual palpation and press the fundus of the uterus down in order to get my finger up into the uterine cavity to separate the placenta. I was therefore obliged to pull the placenta out with the forceps, bit by bit, instead of enucleating it with the finger first. I could not even pull down the cervix with the rulsellum, because I did not want to put any indirect traction on the abdominal wound.

As far as one can judge from the appearance of the fetus at this period of pregnancy, it had been dead some time. The woman has been flowing for some weeks, with a brownish discharge. The placenta does not look like the placenta at term, and it is well for you to see and feel it.

It may seem strange to you that anybody with experience in examining the pelvic organs in women should have made such a mistake. All I can say in extenuation is that the mistake has been made by almost every operator of prominence in the world, of opening the abdomen for some abnormal condition and finding pregnancy. The mistake has been made dozens of times, and it will be made dozens of times in the future, because it is sometimes impossible to diagnosticate pregnancy. In this case the woman had had no symptoms of pregnancy. That matter had been discussed somewhat and thrown out of consideration. It was not suggested to me, and I did not even think of it. The uterus had been curetted within three or four weeks, the sound had been passed, the woman had menstruated shortly before. I think that almost anybody would have made the same mistake, although some might have been in doubt and might have waited, but I anticipated no trouble from the laparatomy, and I therefore performed the operation. Under similar circumstances, I think, I should do the same thing again. The exploratory laparatomy, properly done, can do little harm; there is not one chance in 300 of the woman's dying if it is done under proper conditions. The laparatomy had nothing to do with the miscarriage nor with the rise of temperature. I have seen such a high temperature several times occurring for an hour or two before the abortion begins. It seems to be of nervous origin; it is not septic, because as soon as the abortion gets well under way the temperature goes down and does not recur. I think I have seen some reference to this phenomenon in some journal.

The main lesson which this case teaches is the importance of recognizing the uterus when we find it. I do not want to call attention to anybody else's mistakes, but I must refer to a case which I once heard of. A practitioner thought he had an ovarian cyst to deal with. He opened the abdomen, and without endeavoring to ascertain the nature of the tumor which he came upon, he thrust a trocar into the cyst and then a fetal hand protruded through the trocar opening. He then enlarged the opening, delivered twins, and did Porro's operation. I have no doubt he did it correctly, but the woman died subsequently of peritonitis. I did not blame him for opening the abdomen. I have three or four times opened the abdomen and found pregnancy, but always, except in this case, with some abnormality to cover it up. It seems to me a man ought to be on his guard, not so much against opening the abdomen and finding a pregnant uterus, as against injuring the pregnant uterus because he neglects to make out the condition of affairs by careful examination of the abdominal contents with his hands. Our patient will doubtless make a good recovery.

NOTE.—Recovery followed as anticipated.

